# Metabolism and Nutrition of L-Glutamate and L-Glutamine in Ruminants

**DOI:** 10.3390/ani14121788

**Published:** 2024-06-14

**Authors:** Guoyao Wu, Fuller W. Bazer, Gregory A. Johnson, M. Carey Satterfield, Shannon E. Washburn

**Affiliations:** 1 Department of Animal Science, Texas A&M University, College Station, TX 77843, USA; fuller.bazer@ag.tamu.edu (F.W.B.); carey.satterfield@ag.tamu.edu (M.C.S.); 2 Department of Veterinary Integrative Biosciences, Texas A&M University, College Station, TX 77843, USA; gjohnson@cvm.tamu.edu; 3 Department of Veterinary Physiology and Pharmacology, Texas A&M University, College Station, TX 77843, USA; swashburn@cvm.tamu.edu

**Keywords:** amino acids, glutamate, glutamine, health, metabolism, nutrition, ruminants

## Abstract

**Simple Summary:**

L-Glutamate (Glu) and L-glutamine (Gln) are abundant amino acids in feedstuffs and ruminants. Dietary Gln is extensively utilized by ruminal microbes, but dietary Glu undergoes little catabolism by these microbes because they do not take up extracellular Glu due to the lack of the necessary transporters. Microbial proteins and dietary Glu exit the rumen into the abomasum and then the small intestine, where proteins undergo hydrolysis to release amino acids (including Glu and Gln) and small peptides for transport into enterocytes. Most dietary Gln escapes the underdeveloped rumen of preruminants, instead entering the abomasum and the small intestine. Within the enterocytes, Glu and Gln are extensively oxidized to provide ATP and are actively used to synthesize glutathione and other amino acids (alanine, ornithine, citrulline, arginine, proline, and aspartate), whereas Gln and aspartate are essential for purine and pyrimidine syntheses. Under normal feeding conditions, all diet- and rumen-derived Glu and Gln are extracted by the small intestine and, therefore, do not enter the portal circulation. De novo synthesis plays a crucial role in maintaining the homeostasis of Glu and Gln in the whole body but may be insufficient for maximal growth performance, production (e.g., lactation and pregnancy), and optimal health in ruminants. Dietary supplementation with Glu or Gln can safely improve the digestive, endocrine, and reproduction functions of ruminants and thus augment health and production parameters.

**Abstract:**

Although both L-glutamate (Glu) and L-glutamine (Gln) have long been considered nutritionally nonessential in ruminants, these two amino acids have enormous nutritional and physiological importance. Results of recent studies revealed that extracellular Gln is extensively degraded by ruminal microbes, but extracellular Glu undergoes little catabolism by these cells due to the near absence of its uptake. Ruminal bacteria hydrolyze Gln to Glu plus ammonia and, intracellularly, use both amino acids for protein synthesis. Microbial proteins and dietary Glu enter the small intestine in ruminants. Both Glu and Gln are the major metabolic fuels and building blocks of proteins, as well as substrates for the syntheses of glutathione and amino acids (alanine, ornithine, citrulline, arginine, proline, and aspartate) in the intestinal mucosa. In addition, Gln and aspartate are essential for purine and pyrimidine syntheses, whereas arginine and proline are necessary for the production of nitric oxide (a major vasodilator) and collagen (the most abundant protein in the body), respectively. Under normal feeding conditions, all diet- and rumen-derived Glu and Gln are extensively utilized by the small intestine and do not enter the portal circulation. Thus, de novo synthesis (e.g., from branched-chain amino acids and α-ketoglutarate) plays a crucial role in the homeostasis of Glu and Gln in the whole body but may be insufficient for maximal growth performance, production (e.g., lactation and pregnancy), and optimal health (particularly intestinal health) in ruminants. This applies to all types of feeding systems used around the world (e.g., rearing on a milk replacer before weaning, pasture-based production, and total mixed rations). Dietary supplementation with the appropriate doses of Glu or Gln [e.g., 0.5 or 1 g/kg body weight (BW)/day, respectively] can safely improve the digestive, endocrine, and reproduction functions of ruminants to enhance their productivity. Both Glu and Gln are truly functional amino acids in the nutrition of ruminants and hold great promise for improving their health and productivity.

## 1. Introduction

Both L-glutamate (Glu) and L-glutamine (Gln) are abundant amino acids (AAs) in plant, microbial, and animal proteins [1]. In the whole bodies of sheep and cattle, Glu and Gln are the third and eighth most abundant AAs, respectively. For comparison, the total content of these two AAs, along with other AAs, in feeds [e.g., Bermuda grass, distillers dried grains, and solubles (DDGS)], ruminal microbes, and skeletal muscle proteins is also relatively high, as summarized in Table 1. In postnatal ruminants (e.g., sheep and cattle), including neonates, preruminants, and adults, Gln is the second most abundant free AA in their plasma (after glycine) [2,3], whereas Glu is the most abundant AA in the proteins of skeletal muscle [4]. The high abundance of Glu and Gln in tissues is consistent with their nutritional and physiological significance in animals. However, these two AAs have long been considered nutritionally nonessential in ruminants [5].

Prior to the closure of the esophageal groove and weaning in ruminant animals, the abomasum is the primary recipient of nutrients. This developmental phase is called the preruminant period. Preruminants possess an underdeveloped rumen and, therefore, have similar patterns of metabolism and the utilization of Glu and Gln to those in monogastric animals [6,7,8]. In calves [9] and lambs [10], which are currently weaned at approximately 7 and 3 months of age, respectively, significant proportions of dietary AAs (including Glu and Gln) escape the rumen before weaning [6,11,12], and the oral administration of Glu (0.11 mg/kg BW) or Gln (0.5 g/kg BW) can substantially increase their concentrations in the lumen of the small intestine [13]. In contrast, postweaning ruminants have a large, functional rumen containing many different species of bacteria that extensively utilize most dietary AAs, including arginine and Gln, for the synthesis of microbial proteins [14,15,16,17,18,19,20].

Postweaning, most dietary AAs undergo metabolic transformations in the rumen and do not enter the small intestine intact [4,21,22,23], with the exception of dietary Glu and citrulline [4,19,20]. Of particular note, we recently discovered that the ruminal microbes of cattle [4,19] and sheep [20] have little or no ability to degrade extracellular Glu and citrulline due to negligible or no uptake. Once in the intestines, there is an extensive catabolism of Glu and Gln by the enterocytes and, therefore, little or no rumen-derived or dietary Glu and Gln enters the portal vein [4,21,22,23]. The objective of this article is to review the literature concerning the metabolism and nutrition of Glu and Gln in ruminant species (e.g., cattle, goats, and sheep).

**Table 1:** Composition of amino acids (AAs) in the feeds, ruminal bacterial proteins, plasma, skeletal muscle proteins, and the whole bodies of adult sheep and cattle ^a^.

AAs	AAs in Feeds		AAs in Ruminal		Free AAs in		AAs in Skeletal		AAs in the Whole	
	(g/100 g AAs)		Bacterial Proteins		Plasma (µmol/L)		Muscle Proteins		Body	
			(g/100 g AAs) ^b^				(g/100 g AAs) ^b^		(mg/g AA) ^b^	
	Sheep	Cattle	Sheep	Cattle	Sheep	Cattle	Sheep	Cattle	Sheep	Cattle ^d^
Ala	6.53	8.02	6.74	6.72	182	181	5.52	5.55	66.5	71.2
Arg	5.91	5.18	5.03	5.01	190	121	6.58	6.57	68.0	70.2
Asn	5.13	4.71	5.34	5.36	33	31	4.16	4.18	35.8	36.7
Asp	5.83	6.58	6.74	6.75	11	5.4	5.15	5.16	43.7	44.8
Cys ^c^	1.87	1.61	1.48	1.49	114	132	1.38	1.35	14.6	13.4
Gln	9.02	5.95	5.11	5.13	372	286	5.66	5.64	50.9	49.1
Glu	7.85	10.8	8.02	7.99	61	52	9.35	9.32	83.2	80.2
Gly	4.90	4.93	5.06	5.07	511	347	4.17	4.18	113	113.3
His	2.18	2.28	2.05	2.07	62	67	3.94	3.95	21.2	25.3
Ile	4.20	4.47	5.53	5.51	62	100	5.13	5.15	36.0	28.1
Leu	8.32	8.67	7.67	7.66	107	148	8.34	8.33	69.4	69.3
Lys	4.98	4.66	7.70	7.70	94	104	9.02	9.03	61.0	64.6
Met	1.63	1.79	2.42	2.40	24	27	3.18	3.17	19.0	18.8
Phe	4.90	5.30	5.13	5.16	36	51	4.19	4.18	34.6	36.5
Pro	7.93	4.9	3.67	3.66	156	184	4.06	4.08	85.5	81.5
Ser	5.05	4.56	4.65	4.62	75	67	4.38	4.41	45.2	44.0
Thr	3.81	4.78	5.52	5.57	60	62	4.61	4.59	36.8	40.3
Trp	1.24	1.53	1.39	1.38	39	49	1.26	1.25	11.4	11.4
Tyr	3.73	3.42	4.65	4.63	61	70	3.76	3.75	27.0	25.3
Val	4.98	5.91	6.08	6.11	128	224	5.96	5.94	42.6	39.3
Hyp	ND	ND	ND	ND	41	45	0.20	0.21	34.8	36.7

^a^ except for the composition of amino acids in the whole bodies, data are adapted from Gilbreath et al. [4] for (a) adult Suffolk female sheep (60 to 65 kg) consuming daily 23 g feed (as-fed basis)/kg body weight of a soybean hull-, wheat middling-, and corn-based diet and (b) adult Angus × Hereford steers (mean body weight of 538 kg) consuming daily 14.02 kg (dry matter) of Bermudagrass hay and 0.506 kg (dry matter) of dried distillers grains with solubles. Data on the composition of amino acids in the whole body are adapted from Wu and Li [24]. ^b^ calculations were based on the molecular weights of intact AA for 12-month-old sheep and 12-week-old cattle. ^c^ total cysteine (cysteine plus ½ cystine). ^d^ for 12-week-old cattle. Recalculated from the data of Davis et al. [25] for all AAs except Cys, Met, Trp, and Hyp. In the calf body, the content of Cys, Met, Trp, and Hyp is 13.4, 18.8, 11.4, and 36.7 mg/g protein, respectively, whereas the ratios (g/g) of glutamine/glutamate and asparagine/aspartate are 0.612:1.00 and 0.820:1.00, respectively, as determined by Li and Wu [26]. Hyp, 4-hydroxyproline; ND, not detected.

## 2. Glu and Gln Metabolism in Ruminants

Glu and Gln are interconverted in animal metabolism [1]. They are major AAs in the proteins of milk from ruminants [25,27,28,29] and the whole body [6,30], as well as in uterine and fetal fluids [31,32,33,34,35,36]. Because no rumen-derived or dietary Glu and Gln enters the blood in sheep and cattle under normal feeding conditions (e.g., grasses, forages, and hays), ruminants must be able to synthesize Glu and Gln to account for their high abundance in whole-body proteins and intracellular free pools, as well as their high rates of whole-body catabolism [30]. At present, little is known about the quantitative aspects of Glu and Gln synthesis or catabolism in the whole body of young or adult ruminants. However, there is increasing interest in ruminal Glu and Gln metabolism [14,37,38], as well as hepatic Glu metabolism in growing and fattening cattle [39,40,41]. The following sections describe the role of the rumen and other tissues of domestic ruminants in Glu and Gln metabolism.

### 2.1. Glu and Gln Synthesis in Ruminants

#### 2.1.1. Glu and Gln Synthesis in the Rumen

**Figure 1:**
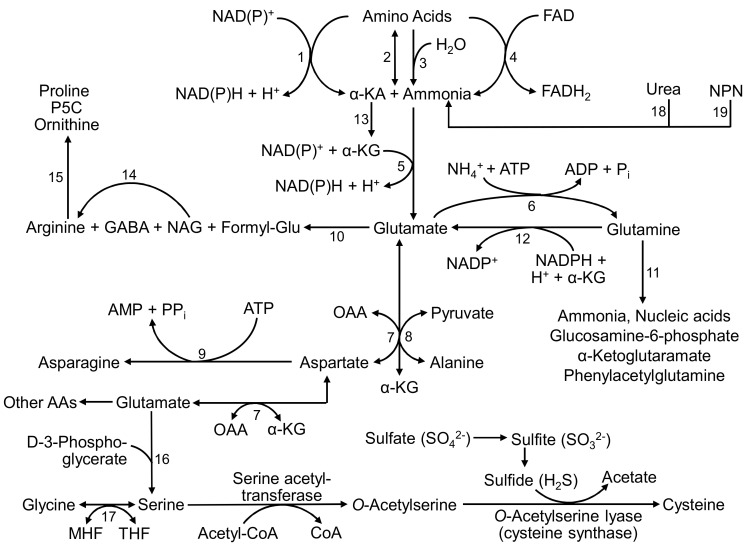
**Production and utilization of ammonia by microorganisms in the rumen of ruminants.** GABA, γ-aminobutyrate; Formyl-Glu, formylglutamate; α-KA, α-ketoacids; α-KG, α-ketoglutarate; NAG, N-acetylglutamate; P5C, pyrroline-5-caroxylate. The enzymes that catalyze the indicated reactions are: (1) amino acid (AA) dehydrogenases; (2) AA transaminases; (3) AA deaminases; (4) AA oxidases; (5) glutamate dehydrogenase; (6) glutamine synthetase; (7) glutamate-oxaloacetate transaminase (aspartate transaminase); (8) glutamate-pyruvate transaminase (alanine transaminase); (9) asparagine synthetase; (10) The syntheses of NAG, GABA, P5C, and formyl-Glu from glutamate are catalyzed by NAG synthase, glutamate decarboxylase, γ-glutamyl kinase plus glutamyl semialdehyde dehydrogenase, and complex enzymes, respectively; (11) a series of enzymes required in multiple pathways; (12) glutamate synthase (also known as NADPH-dependent glutamine:α-ketoglutarate amidotransferase; glutamine + 2 α-ketoglutarate + NADPH + H^+^ → 2 glutamate + NADP^+^); and (13) conversion of α-ketoacids to α-ketoglutarate via various reactions; (14) the enzymes for converting NAG into arginine; (15) arginase, ornithine aminotransferase, and P5C reductase; and (16) enzymes for converting D-3-phosphoglycerate and glutamate into serine; (17) serine hydroxymethyltransferase; (18) urease; and (19) enzymes for converting non-protein nitrogen into ammonia. MTF, *N*^5^-*N*^10^-methylene tetrahydrofolate; NAG, N-acetyl-glutamate; OAA = oxaloacetate; NPN, non-protein nitrogen; P5C, pyrroline-5-carboxylate; THF, tetrahydrofolate. Other AAs include His, Lys, Phe, and branched-chain AAs. Adapted from Wu [30].

Bacteria in the rumen are capable of synthesizing Glu from ammonia and α-ketoglutarate (α-KG) via Glu dehydrogenase, and Glu is subsequently amidated with ammonia to form Gln by Gln synthetase (Figure 1). These two enzymes are crucial for assimilating ammonia in ruminants. The ammonia used for these synthetic reactions is derived from the degradation of protein, AAs, and non-AA nitrogen (e.g., urea, amines, and nucleic acids) in diets, as well as urea and AAs from the saliva and blood [42]. The sources of α-KG are the catabolism of carbohydrates, propionate, and AAs. Ruminal microbes also use intracellular AAs [e.g., branched-chain AAs (BCAAs)] to form Glu and Gln via complex pathways (Figure 1). The active generation of both Glu and Gln in the rumen plays an important role in the synthesis of microbial protein, while reducing the amounts of carbon skeletons for methane production. The microbial protein subsequently enters the abomasum and the small intestine, where hydrolysis releases Glu and Gln as well as small peptides [43]. Thus, in the rumen, intracellularly generated Glu and Gln can be used directly for synthetic pathways, thereby increasing the energetic efficiency of dietary AAs for the growth, reproduction, and lactation in ruminants [4].

#### 2.1.2. Glu and Gln Synthesis in Extra-Ruminal Tissues

There is an extensive catabolism of Glu and Gln by the enterocytes and, therefore, all or nearly all of the rumen-derived or dietary Glu and Gln do not enter the portal vein [4,21,22,23]. Under normal feeding conditions, abomasal-infused Glu or Gln (e.g., 5 g/day) does not enter the portal vein in adult sheep [21]. These findings indicate that the rumen-derived (microbial protein) or dietary Glu and Gln are completely sequestered or utilized by the small intestine of ruminants in the first pass, as reported for monogastric animals [44]. However, Glu is one of the most abundant AAs in the proteins of ruminant bodies [24] and feeds [4,26,45]. Of note, Glu is the most abundant AA in the proteins of skeletal muscle (Table 1), which represents 45% of the total BW [46], whereas Gln is one of the most abundant α-AAs in the free plasma pool and muscle proteins [47]. Furthermore, immunocytes [48] and erythrocytes [1] in the blood of ruminants actively use Gln mainly via phosphate-activated glutaminase and glutamine:fructose-6-phosphate transaminase pathways, respectively. Clearly, the de novo synthesis of Glu and Gln must occur in the body for the production of polypeptides and proteins.

In support of an important role for BCAAs in the generation of Glu and Gln in ruminants, Wijayasinghe et al. [49] reported that leucine, isoleucine, and valine were actively catabolized in ovine skeletal muscle to generate (a) Glu by BCAA transaminase and then (b) Gln by Gln synthetase. Additionally, in sheep, the liver [50,51,52], skeletal muscle [53,54], white adipose tissue [55], and placenta [55,56,57] also synthesize and release Gln, as do the kidneys [57,58]. In addition, the skeletal muscle of sheep [53] releases Gln and Ala under both fed and starved conditions. Similar results were reported for cattle such as steers and cows [22,23,59]. The liver of cattle also releases Glu under normal feeding conditions [52]. The immediate precursors of Gln in these tissues are Glu and ammonia, which are derived from the blood and intracellular metabolism [1]. Furthermore, the bovine mammary epithelial cells produce a large amount of Glu and Gln from BCAAs plus α-KG to support the synthesis of milk proteins [60], in agreement with reports for the mammary tissue of lactating sows [48].

The endogenous synthesis of Glu and Gln is essential for maintaining their homeostasis, as well as optimal growth, development, and productivity (e.g., lactation and gestation) in ruminants consuming adequate energy, dry matter (DM) and crude protein (CP) [47,51,61], as reported for nonruminant mammals [30,62]. Based on the knowledge of nitrogen metabolism [30,58,63,64], it can be estimated that approximately 31% and 30% of dietary CP is used for the whole-body synthesis of Glu and Gln, respectively, in adult, non-pregnant, and non-lactating sheep (Table 2). Most of the dietary protein consumed by adult ruminants is used for the production of Glu plus Gln. Under conventional feeding conditions (e.g., grazing, feedlot, or intensive management), the Glu- or Gln-synthetic capacity may not be sufficient for maximal production performance or maximal feed efficiency, particularly under the conditions of early lactation [65,66], heat stress [67,68], infections [69,70,71], acid-base imbalances [47,72], and intestinal dysfunction [73].

### 2.2. Glu and Gln Catabolism in Ruminants

#### 2.2.1. Glu and Gln Catabolism in the Rumen

In preruminants, the rumen is not an active site for the catabolism of dietary AAs (including Glu and Gln) [12,61,74]. After weaning, when the rumen has developed and is functional, it plays an important role in fermenting dietary protein and AAs. Ruminal microbes have long been considered capable of extensively degrading all dietary AAs [16,75]. Interestingly, we recently identified the active degradation of extracellular Gln but little degradation of extracellular Glu by ruminal microbes from adult cattle [14,19] and sheep [20]. Specifically, in our in vitro experiments, whole rumen fluid (3 mL) from adult steers or sheep was incubated at 37 °C with 5 mM Gln or 5 mM Glu for 0.5, 1, 2, or 4 h, and 50 µL samples were collected at predetermined time points for AA analyses. Our results revealed the extensive hydrolysis of Gln into Glu, but little degradation of extracellular Glu by ruminal microbes during a 4 h period of incubation. This finding can be explained by a high rate of the uptake of extracellular [^14^C]Gln but little uptake of [^14^C]Glu by the ruminal microbes. In our in vivo experiments, after Gln was orally administered to adult (non-pregnant and non-lactating) steers [14,19] and sheep [20], Glu rapidly accumulated in the ruminal fluid, but extracellular Glu did not undergo significant catabolism. Thus, large amounts of the rumen-derived (microbial) or dietary Glu can enter the abomasum and small intestine (Table 2). In support of this concept, dietary supplementation with unprotected Glu (i.e., without any encapsulation) in adult steers can effectively increase its availability to the small intestine, thereby regulating gut motility and starch digestion [76]. Therefore, in the rumen, extracellular Glu has a very different metabolic fate than intracellular Glu. Such intra-ruminal compartmentation of Glu metabolism has important implications for the use of crystalline Glu in improving the intestinal health and function of ruminants.

#### 2.2.2. Glu Catabolism in Extra-Ruminal Tissues

In the small intestine of ruminants, the apical membranes of enterocytes possess the following: (a) excitatory AA carrier 1 (EAAC1) as the major transporter for taking up dietary Glu, and (b) a peptide transporter 1 (PepT1) that transports specifically di- and tri-peptides [77]. In addition, enterocytes express high activities of enzymes that hydrolyze small peptides and degrade Glu (primarily via transamination). Studies in beef cattle [22,59,78], dairy cows [23], and sheep [55,79,80] have all shown that the mucosa of the small intestine rapidly takes up and extensively degrades Glu or Glu-containing small peptides present in its lumen. Intestinal Glu catabolism generates CO_2_, alanine, aspartate, ornithine, citrulline, arginine, proline, pyruvate, and lactate as the primary products [21,74,81], while providing a large amount of energy for utilization by enterocytes [22]. Of note, in ruminants, as in most of the omnivorous mammals, the formation of ornithine, citrulline, arginine, and proline from Glu occurs almost exclusively in enterocytes [74,82]. The conversion of Glu into arginine [the precursor of nitric oxide (a major vasodilator)] is quantitatively important to support the productivity of ruminants, such as growth, reproduction, and lactation [74,83]. In addition, proline is required for the production of collagen (the most abundant protein in the body) [1]. Furthermore, Gln and aspartate are essential for purine and pyrimidine syntheses. Thus, due to its extensive use by the enterocytes, the Glu that flows from the abomasum into the small intestine does not enter the portal vein of adult sheep [21], steers [52,59], or dairy cows [23,84].

As reported for non-ruminants including pigs [85], the small intestine of sheep [21,86] and cattle [23,87,88] does not take up Glu from the arterial blood due to the lack of the expression of Glu transporters in the basolateral membranes of the enterocytes. Nonetheless, Glu in the blood is rapidly and extensively oxidized in ruminants (including dairy cows, sheep, and goats), with CO_2_ and glucose being the major metabolic products [57,84,89,90]. For example, 40% and 43% of intravenously administered [U-^14^C]Glu appeared as ^14^CO_2_ within 3 h in lactating goats [90] and lactating cows [84], respectively. Furthermore, in lactating goats, 1.13–2.34%, 0.62–1.38%, 0.42–1.90%, and 0.30–0.32% of intravenously administered [U-^14^C]Glu was recovered in lactose, casein, fat, and albumin, respectively, within a 48-h period [90]. Likewise, in lactating cows, 6.09–7.29%, 3.07–5.07%, 1.03–1.33%, and 0.44–0.84% of intravenously administered [U-^14^C]Glu was recovered in lactose, casein, fat, and albumin, respectively, within a 48-h period [89]. Thus, Glu in the blood is differentially used by different organs. This illustrates the complex compartmentation of whole-body Glu catabolism in animals (including ruminants), and also indicates an important role for microbial protein synthesis (as the major source of Glu) in gut nutrition and metabolism under normal feeding conditions.

With the exception of periportal hepatocytes and red blood cells [91], the extra-intestinal tissues of ruminants also express EAAC1 as the major transporter for taking up extracellular Glu [37,55,77]. As in the liver of non-ruminants [1], perivenous hepatocytes (but not periportal hepatocytes) take up Glu from the blood in ruminants and this activity decreases with age [39]. The turnover rate of Glu is 0.596 mg/min/kg BW (i.e., 0.243 mmol/h/kg BW) in fed adult sheep (75 kg BW), with CO_2_ and glucose production accounting for 57.7% and 11.3% of the metabolized Glu, respectively [64]. Under normal feeding conditions, the liver of ruminants, including lactating cows (Table 3), beef cattle [52], and sheep [92], has a net release of Glu likely due to hepatic AA metabolism for Glu synthesis. In extra-intestinal tissues, Glu is used for the synthesis of not only protein and polypeptides but also Gln, glutathione, and low-molecular-weight substances (e.g., γ-aminobutyrate) with enormous physiological importance [1]. There is evidence that the skeletal muscle and kidneys of cattle [50] and sheep [57] actively extract Glu from the arterial blood to support Gln and protein synthesis, as well as ammoniagenesis and acid/base balance.

#### 2.2.3. Gln Catabolism in Extra-Ruminal Tissues

In the small intestine of ruminants, the apical membranes of enterocytes possess (a) neutral AA transporter B^0^AT1 and (b) sodium-dependent neutral AA transporters (SNAT) 1 and 2 as major transporters for absorbing free Gln from the lumen of the gut [39,55,77,93,94]. As noted previously, the apical membranes of enterocytes have the highly active PepT1 that transports di- and tri-peptides, including those containing Gln. Much evidence shows that, in ruminants, Gln is extensively catabolized by the small intestine (primarily via phosphate-activated glutaminase) during first-pass metabolism such that rumen-derived and dietary Gln do not enter the portal circulation (Table 2). These animals include growing beef cattle [22,59,78], lactating (non-pregnant) dairy cows [87], periparturient dairy cows (12 days prepartum and days 4–29 postpartum) [95], adult steers [96], adult (non-pregnant and non-lactating) sheep [21,58], and young (35-kg) ram lambs [86]. Although the small intestine accounts for 2–3% of the BW, the splanchnic utilization of Gln accounts for 45–70% of whole-body Gln flux in ruminants [51,87,95]. Interestingly, Gln is a major anaplerotic substrate in the duodenal mucosal cells of cattle [7] and sheep [80], as well as the hepatocytes of dairy cows [84]. The oral administration of 50 g Gln to adult steers twice daily (i.e., 100 g Gln/day) [14,19] or 5 g Gln once to adult, non-pregnant, and non-lactating ewes [20] did not affect the concentrations of Gln in their plasma. Similarly, Plaizier et al. [97] reported that the intra-abomasal infusion of 100 g Gln/day to lactating cows did not influence the concentrations of Gln in plasma. The major products of Gln in the mucosa of the ruminant small intestine include not only Glu and its metabolites (as noted previously) but also ammonia and aminosugars [4]. Table 4 summarizes age-dependent changes in the catabolism of Gln for the production of CO_2_, ornithine, citrulline, and arginine by bovine and ovine enterocytes.

The small intestine of all animals has an upper limit of the capacity to utilize dietary Gln. Thus, as in monogastric animals [30], when excessive Gln in the lumen of the small intestine (e.g., achieved through the intragastric infusion of large amounts of Gln or protein) exceeds the capacity of the small intestine for Gln utilization, some of this AA escapes intestinal catabolism and enters the portal circulation. Consistent with this idea, Meijer et al. [98] reported that the intra-abomasal infusion of 300 g Gln/day to lactating cows (DM intake = 20.0–22.3 kg/day) increased the concentrations of Gln and urea in plasma by 60% and 44%, respectively. This was replicated by the study of Doepel et al. [87], in which the intra-abomasal infusion of 300 g Gln/day to lactating cows (DM intake = 18.1 kg/day) increased the concentrations of Gln and urea in plasma by 44% and 23%, respectively. Such a high dose of Gln may not be ideal for the intestinal absorption of other AAs, as it substantially reduced the concentrations of glycine (−18%), tryptophan (–21%), threonine (–20%), and tyrosine (–22%) in the arterial plasma [87], likely due to competitive inhibition of their transport by enterocytes [1]. Compared with the dose of 0 g Gln/day, the intra-abomasal infusions of 200 and 300 g Gln/day to lactating cows (DM intake = 21.5 kg/day) dose-dependently increased the concentrations of Gln in plasma by 23% and 33%, respectively [97].

In addition to enteral Gln, the basolateral membranes of mammalian enterocytes possess Gln transporters for taking up Gln from the arterial blood [93]. In the postabsorptive state, Gln is the only AA in the blood that is taken up by the intestinal mucosa, where Gln is the major source of intracellular Glu. In contrast, the conversion of Glu into Gln is limited in the small intestine of both neonatal and adult ruminants. Because the mucosa of the small intestine critically depends on enteral Gln for survival and antioxidative responses, rumen-derived Gln (in the form of microbial protein) is crucial for the health and function of the small intestine under normal feeding conditions.

In ruminants, Gln in the blood can be taken up by extra-intestinal tissues, such as the liver [11], skeletal muscle [50,99], kidneys [70,71], mammary tissue [60], lymphocytes [48], and macrophages [100]. As in non-ruminants, periportal hepatocytes extract Gln from the blood to produce Glu and urea in ruminants [87,101]. In fed adult sheep and 3-day-fasted sheep (50–60 kg BW), the rates of whole-body Gln catabolism are 12.2 and 11.5 mmol/h, respectively; or 0.222 and 0.209 mmol/h/kg BW, respectively [58]. Interestingly, glucose synthesis accounts for 17% and 20% of the Gln utilized by adult sheep in the fed and fasted states, respectively [58]. Similar results were reported for dairy cows, with CO_2_ and glucose being the major metabolic products of Gln [84]. For example, 27% of the intravenously administered [U-^14^C]Gln appeared as ^14^CO_2_ within 3 h in dairy cows [84]. Thus, under normal feeding conditions, the liver of ruminants, including lactating cows (Table 3) and adult (non-pregnant and non-lactating) sheep [92], has a net uptake of Gln as a substrate for gluconeogenesis. The hepatic uptake of Gln increased under inflammatory conditions [71] to support the metabolism of Kupffer cells (also known as stellate macrophages), as well as the synthesis of glucose and acute phase proteins. In response to acidosis, the liver of sheep extracts less Gln, compared with normal sheep [102], and this aspect of Gln metabolism differs from the liver of non-ruminants, which releases Gln in response to acidosis [103].

It is also of interest that the abomasal infusion of Gln does not influence glucose metabolism in the portal-drained viscera of ruminants, including sheep [69] and cattle [97]. Likewise, the abomasal infusion of 1.5 kg glucose/day has no effect on the splanchnic metabolism of Gln and Glu and does not increase the release of alanine from the portal-drained viscera in ruminants such as sheep [104,105], periparturient dairy cows [95], and lactating dairy cows [106]. This aspect of Gln and glucose metabolism in the portal-drained viscera of ruminants also differs from that in non-ruminants, where glucose provision reduces intestinal Gln catabolism and vice versa [1,30]. It is possible that the rate of glucose catabolism by the small-intestinal mucosa is much lower in ruminants than in nonruminants and that dietary Gln is not a major glucogenic substrate in ruminants because of extensive catabolism to CO_2_ by the small intestine [4,30].

### 2.3. Developmental Changes in Glu and Gln Metabolism in Postnatal Ruminants

We are not aware of any studies of age-dependent changes in whole-body Glu or Gln metabolism in postnatal ruminants. Enzymatic data (expressed per g wet tissue) from studies with sheep suggest developmental changes in Glu and Gln metabolism in a tissue-specific manner [107]. First, in the liver, the activities of glutamine synthetase, phosphate-activated glutaminase, and glutamate dehydrogenase increase during the suckling period (days 0–43 after birth) and thereafter decrease during the subsequent postweaning period (70 days). Second, in the kidney cortex, the activities of glutamine synthetase and glutamate dehydrogenase increase gradually between days 0 and 113 after birth, whereas the activities of phosphate-activated glutaminase follow a similar pattern to the hepatic enzyme. Third, in skeletal muscle, the activities of glutamine synthetase and glutamate dehydrogenase follow a comparable pattern to those seen in the hepatic enzymes, whereas the activities of phosphate-activated glutaminase decrease gradually between days 0 and 113 after birth. Fourth, compared with young sheep (~6 months of age), the activities of glutamate/oxaloacetate transaminase in adult (3- to 4-year-old non-pregnant and non-lactating) sheep increase in skeletal muscle but do not change in the liver or kidneys [108]. Furthermore, the rates of Gln oxidation as well as the synthesis of ornithine, citrulline, and arginine in both bovine and ovine enterocytes decrease gradually between birth and 24 months of age [74].

## 3. Glu and Gln Nutrition in Ruminants

Both Glu and Gln are major metabolic fuels in the small intestine of ruminants, as noted previously. In addition, Glu is essential for the synthesis of GSH (a major antioxidant) in animals, thereby protecting them from oxidative stress [109]. Furthermore, Gln serves not only as a building block of tissue protein but also as a signaling molecule to stimulate mTOR and protein synthesis in ruminants [72,110,111]. Gln is essential for sperm production and function [112,113,114], embryonic development [115,116,117,118], fetal growth [72], lactation [119], the regulation of acid-base balance [47], intestinal health and function [69,120], and skeletal muscle growth [50] in ruminants. At present, the National Research Council (NRC) has not established dietary requirements for preruminants or ruminants (e.g., calves, beef cattle, dairy cows, lambs, and ewes) for Glu or Gln [75,121]. However, there is evidence that preruminants, as well as adult sheep, goats, dairy cows, and cattle, require dietary Glu (Table 5) and Gln (Table 6) supplementation for maximal growth and production performance, as well as for optimal (particularly intestinal) health and function, as detailed in the following sections [122,123,124,125,126,127,128,129,130,131,132,133,134,135,136,137,138,139,140,141,142,143,144,145,146,147,148,149,150,151,152,153,154,155,156,157,158,159,160,161,162,163,164,165,166]. This represents a paradigm shift in ruminant nutrition.

### 3.1. Glu Nutrition in Ruminants

#### 3.1.1. Glu Nutrition and Feed Intake in Calves

Oltjen et al. [122] reported that dietary supplementation with 0.3% Glu for 7 days to steer calves fed a urea-based purified diet (providing adequate starch, fiber, minerals, choline, vitamins A and D, and fatty acids, as well as 97% of total nitrogen from urea) increased the concentrations of butyrate plus longer-chain fatty acids in ruminal fluid by 41%, without affecting those of acetate, propionate, ammonia, or proton. This finding indicated a novel role for dietary Glu in modulating the fermentation of carbohydrates by ruminal microbes. Additionally, it has long been known that Glu stimulates food consumption by non-ruminants such as humans and pigs [146]. In nutrition, a low-cost source of Glu is monosodium glutamate (MSG). Because MSG also confers good taste to certain mammals (e.g., humans and pigs), this substance may enhance feed intake by young ruminants. Waldern and Van Dyk [123] conducted the following two series of experiments with early-weaned calves fed a high-quality diet to test this hypothesis.

In Experiment 1, dairy calves were fed 2.3-kg fresh whole milk twice daily and received a starter diet supplemented with 0 or 0.2% MSG between 0 and 21 days of age [123]. The calves were weaned at 3 weeks of age to a sun-cured alfalfa-based complete diet (containing 18% CP) supplemented with 0 or 0.2% MSG until 84 days of age. During the third week of age, preweaning calves receiving the MSG-supplemented starter diet consumed 321% more solid feed than the non-supplemented group. During the fourth, fifth, and sixth weeks of age (weeks 1, 2 and 3 postweaning), postweaning calves fed the MSG-supplemented diet consumed 248, 107 and 40% more solid feed than the non-supplemented group, respectively. Overall, dietary supplementation with MSG enhanced feed intake by 79% between weeks 1 and 6 of the trial when compared with the control group, likely because the binding of MSG to bovine taste receptors in the tongue stimulates the central appetite regulation center [147]. Interestingly, the marked increase in feed intake did not promote the BW gain of MSG-supplemented calves. It is possible that the basal diet did not provide sufficient amounts of certain nutrients, which limited the growth response to dietary MSG supplementation. Alternatively, perhaps the small number of calves used in the experiment (*n* = 10/group) was not sufficient to detect a change in BW between the control and MSG-supplemented calves. Furthermore, no significant differences in feed intake were detected between the control and MSG-supplemented calves during weeks 7 and 12 of the trial, and the authors offered no adequate explanation for this observation.

In Experiment 2 of Waldern and Van Dyk [123], dairy calves were fed 2.3-kg fresh whole milk twice daily and received a starter diet supplemented with 0 or 0.2% MSG between 0 and 28 days of age. The calves were weaned at 4 weeks of age to a sun-cured alfalfa-based complete diet (containing 18% CP) supplemented with 0 or 0.2% MSG. Compared with the control group, dietary supplementation with MSG numerically increased weekly feed intake by 76, 58, 19, and 23% during weeks 3, 4, 5, and 6 after birth, respectively. These changes were statistically insignificant possibly because only a small number of calves was used in the experiment (*n* = 10/group). Of note, calves in the control group lost more weight than MSG-supplemented calves (91 vs. 45 g/day per animal) in the first week after weaning. Because nutrients other than Glu might also limit protein synthesis in early-weaned calves, daily weight gain or feed efficiency did not differ between the control and MSG-supplemented calves during the entire 4-week experimental period. Again, as in Experiment 1, Experiment 2 might not have included a sufficient number of animals to detect growth responses of calves to dietary MSG supplementation, because the authors did not appear to do a statistical power analysis to determine the required sample size for the study.

Ahangarani et al. [148] reported that increasing the content of Glu in a milk replacer diet (containing 24.8% CP and 19.1% fat) from 4.94% to 5.14% (on a DM basis) through Glu supplementation did not affect the feed intake or growth performance of male Holstein calves between 3 and 59 days of age. Likewise, the concentrations of all proteinogenic AAs, ornithine, citrulline, and urea in plasma did not differ between the control and Glu-supplemented calves [148]. Such a dose of Glu supplementation (i.e., 0.2% of the diet on a DM basis) [148] may be too low to elicit nutritionally or physiologically significant responses in calves. Of note, this study lacked an isonitrogenous control group. In addition, it is possible that Ahangarani et al. [148] did not correctly express the dietary content of Glu, which might have included Glu plus Gln after the acid hydrolysis of feed proteins in the laboratory analysis.

#### 3.1.2. Glu Nutrition and Feed Intake in Lambs

Galgan and Russell [124] reported that neonatal lambs, beginning at 18 days of age, consumed more of a high-concentrate diet supplemented with 0.5% MSG than the unsupplemented group. Interestingly, when the basal diet consisted of equal parts of lucerne and concentrates, supplementation with 0.5% MSG increased both the feed intake and live-weight gains of lambs from 90 to 135 days of age, in comparison with the unsupplemented group [124]. These results indicate the beneficial effects of dietary MSG supplementation on growth performance and feed efficiency in young lambs. Similarly, Li et al. [129] reported that dietary supplementation with 3 g Glu to growing male lambs (Hu sheep; with an initial mean BW of 17.74 kg) for 90 days enhanced rumen fermentation, antioxidative capacity, and growth performance.

#### 3.1.3. Glu Nutrition and Feed Intake in Adult Sheep

Colucci and Grovum [125] determined the effects of dietary MSG supplementation on feed intake by adult sheep with or without esophageal fistulas for a period of 64 days. Intakes of fine-ground loose straw (25 g/30 min) by sheep with esophageal fistulas were much lower than those of ground and pelleted straw (711 g/30 min). Dietary supplementation with 0.5–4% MSG to fine and coarse ground straw increased the feed intake of sheep with esophageal fistulas by 146 and 164%, respectively. These findings indicated that adding MSG to low-quality diets could improve their palatability. Similar effects of MSG supplementation on feed intake were observed when the sheep with esophageal fistulas were fed straw pellets. Furthermore, when ammoniated barley straw supplemented with 1% MSG was fed to normal sheep (without esophageal fistulas), DM intake increased by 10%. Similarly, dietary supplementation with 0.5 and 4% MSG (air-dry feed basis) increased the intakes of the pelleted lucerne by 16 and 40%, respectively [149]. Collectively, these results indicate that the intake of either straw or lucerne by sheep may be enhanced by adding MSG to pellets or cubes.

#### 3.1.4. Glu Nutrition in Rams

Glu stimulates the secretion of gonadotropin-releasing hormone (GnRH) from GnRH neurons within the hypothalamus [126]. Meza-Herrera et al. [126] conducted an experiment to test the hypothesis that Glu might improve the quality and quantity of semen in young rams under long-day photoperiods in northern Mexico. Dorper rams received the intramuscular administration of either Glu (7 mg/kg BW) or saline (control) every 3 days for 28 days. Compared with the control group, the Glu treatment increased sperm concentrations in semen by 51% without affecting ejaculation latency, seminal volume, sperm motility, or the percentage of live sperm. This finding indicates a role for Glu in promoting spermatogenesis in rams (a physiological event subject to control by ambient temperatures [150]) and may have important implications for enhancing sperm quantity under cold or heat stress conditions in this and other mammalian species [164].

#### 3.1.5. Glu Nutrition in Female Goats

There are reports that Glu may affect reproductive function in female goats. Torres-Moreno et al. [127] found that the intravenous administration of Glu (7 mg/kg BW) twice weekly (Monday and Friday) between mid-June and late September in northern Mexico enhanced the onset of puberty in female goats without affecting body condition scores or the concentrations of insulin, urea and glucose in plasma. Similar results were obtained for prepubertal female goats receiving the intravenous administration of Glu (7 mg/kg BW) twice weekly between early June and early November [151]. There is also evidence that neither live weight nor body condition differed between the control and Glu-treated (0.175 mg/kg) adult cyclic goats but the number of antral follicles (3.4 vs. 2.1) and ovulation rates (2.5 vs. 1.5) were greater in the Glu group than in the control group [152]. Likewise, results of recent studies indicated that the intravenous administration of Glu to female goats increased the ovulation rate and the number of antral follicles and advanced the onset of early puberty while promoting the return to the reproductive cyclicity of goats in seasonal anestrus [153,154]. These findings implicate a role for Glu in modulating the reproductive performance of female goats. In support of this view, Soares et al. [131] reported that dietary supplementation with MSG (1 g/kg BW) for 23 days increased feed intake, ruminal movement, the frequency of rumination, glutathione peroxide activity in serum, the pulsatility of luteinizing hormone, the number of ovarian follicles, and intraovarian blood perfusion in adult female goats fed a total mixed ration. Thus, as a nutritional supplement, Glu may have a positive effect on improving the reproductive performance of female goats. Because physiological concentrations of Glu in the blood do not cross the blood/brain barrier into the brain [155], it is unclear how the intravenous infusion of this AA increases the release of reproduction-related neuropeptides.

#### 3.1.6. Glu Nutrition in Beef Cattle

Brake et al. [76] determined the effects of the continuous duodenal infusion of raw cornstarch (1391 g/day) with 0 (control) or 133 g Glu/day for 6 days on small-intestinal starch digestion in steers (259-kg BW) fed a low-starch soybean hull-based diet. Compared with the control, duodenal infusion of Glu increased small-intestinal starch digestion and absorption by 21% without affecting the concentrations of cholecystokinin and glucose in plasma. The authors reported that the beneficial effect of the duodenal infusion of Glu was similar to that of casein (400 g/d), suggesting a role of Glu in regulating ruminant intestinal digestion and health. In contrast, the results of a more recent study indicated that the long-term (42-day) duodenal infusion of raw cornstarch (1460 g/day) with 0 (control) or 121 g Glu/day did not affect small-intestinal starch digestion in younger steers (with a mean initial BW of 179-kg BW) fed a low-starch soybean hull–based diet [156]. The reason for this discrepancy is unknown but it is possible the effect of Glu is influenced by the physiological state (e.g., age and BW) of cattle, as well as the dose (g/kg BW) and length (days) of Glu and starch administration.

#### 3.1.7. Glu Nutrition in Dairy Cows

Dairy cows generally have a low appetite during the first few weeks of lactation, leading to reduced milk production. Thus, it is a logical strategy to supplement their diets with MSG as a flavor enhancer. In this regard, it is noteworthy that Nakanishi et al. [128] reported that dairy cows showed a moderate preference for tap water containing 0.08% MSG and the strongest preference for tap water containing 0.32% MSG. Based on this finding, an industrial MSG by-product, which contained 4.8% Glu (on a DM basis), could replace 5, 10 and 15% soybean meal in the basal diet for lactating dairy cows without negatively affecting their lactation performance, as compared with the control diet containing 25% soybean meal [157]. Of note, dietary supplementation with 5, 10 and 15% MSG by-product (providing 0.24, 0.48, and 0.72% supplemental Glu in the diet) reduced feed cost by 2.9–17.3% and increased the profits from milk production by 15, 22, and 33%, respectively [157]. Extracellular Glu may regulate the growth of ruminal microbes in dairy cows through yet unknown regulatory mechanisms. Specifically, adding 3.4 mM Glu to the culture medium (containing 19.5 mM urea as the sole nitrogen source) for 6 h stimulated the growth of mixed ruminal microbes from dairy cows [158]. Similarly, supplementing 0.89 mM Glu to culture medium (containing 2.68 mM ammonium sulfate as the sole nitrogen source) between the initial and middle periods of the exponential growth phase stimulated the growth of mixed ruminal microbes from dairy cows [159]. Of note, 1 mM Glu can prevent the inhibitory effect of 1 mM threonine on the growth of mixed ruminal microbes (isolated from dairy cows) cultured in a medium (containing 2.68 mM ammonium sulfate as the sole nitrogen source) [160]. These results indicate a potential role for extracellular Glu in modulating the growth of ruminal microbes.

In contrast, Dann et al. [161] reported that adding 40 or 80 g/day Glu (as chemical grade MSG equivalent to 0.16% and 0.32% of dietary DM, respectively) per cow to corn silage-, alfalfa grass silage-, ground corn-, and soybean meal-based diets (containing 17% CP on a DM basis) did not affect the digestibilities of organic matter, DM, or fiber and non-starch carbohydrates; the production of short-chain fatty acids by ruminal microbes; or fermenter pH; but dose-dependently decreased CP digestion in the rumen and microbial growth (Experiment 1). These authors [161] suggested, despite a lack of direct evidence, that Glu reduced the utilization of feed protein by ruminal microbes possibly by acting as an inhibitor of ruminal proteases or preventing the uptake of peptides or AAs by ruminal bacteria. However, this proposition is not consistent with the findings that adding 5 mM Glu to bovine or ovine ruminal fluids (containing microbes) had no effect on ammonia production [14,20]. In Experiment 3 (a cross-over design of two periods) of Dann et al. [161], dairy cows were fed for 28 days in each period corn silage-, alfalfa grass silage-, corn meal-, soybean meal-, Canola meal-based diets (19% CP on a DM basis) supplemented with 0 or 80 g/day Glu [as a Food Chemicals Codex (FCC)-grade MSG product containing 99% MSG) per cow. The supplemental Glu was equivalent to 0.33% of the dietary DM. These authors found that the Glu supplementation did not affect actual milk yield, 35 g/kg fat- or energy-corrected milk yield, milk protein or lactose output, the concentration of urea in milk, or the body condition score of cows, while reducing their BW gains (16.9 vs. 5.8 kg over 4 weeks), as compared with the control group without Glu supplementation (Experiment 3) [161]. The reason for such a negative effect of dietary Glu supplementation on the metabolism of cows is unknown, but there is the possibility that the MSG product used in the studies might have contained an unknown toxic substance(s). Unfortunately, Dann et al. [139] did not assess either the purity of the MSG product or the content of AAs (including Glu) in the basal diets. The authors’ adverse in vivo results should not be interpreted to indicate that dietary supplementation with a small amount of Glu (approximately 0.3%) is toxic to dairy cows. In this regard, it is noteworthy that Dann et al. [161] acknowledged that the change in the BW of cows was not biologically meaningful.

We are aware of a report by Nombekela et al. [162] that dietary supplementation with 0.15% MSG reduced the feed intake of dairy cows in early lactation by 25%, compared with the control group without MSG in the diet. However, care should be taken when interpreting this result for the following reasons. First, only one dose of MSG was used in the study, and the supplemental dosage might not have been optimal for cows fed the basal diet. Second, the authors did not verify, by actual chemical analysis, whether or not the complete diet contained a correct amount of MSG. Third, the authors did not provide any information about the chemical purity of the MSG product used, and it is possible that a contaminating substance might have inhibited feed intake by cows. Fourth, the number of cows used in the study (*n* = 6) was too small to allow for drawing a definite conclusion. In contrast, Hisadomi et al. [130] reported that dietary supplementation with rumen-protected MSG (1.54% and 1.14% Glu during prepartum and postpartum periods, respectively) increased digestive capacity and feed intake while reducing body fat and protein mobilization after calving, as demonstrated in lactating sows receiving dietary Glu supplementation [163].

#### 3.1.8. Safety of Glu Supplementation in Ruminants

The safety of Glu supplementation in ruminants depends on the route of administration (e.g., enteral or intravenous), the dietary intake of all AAs and other nutrients, as well as the physiological or pathological conditions of the animals. Regulatory guidelines should be followed to identify the responses of ruminants to graded supplemental doses and, therefore, the No Observed Adverse Effect Levels (NOAEL) [165]. As summarized in the following paragraphs, the current literature indicates that Glu is safe for use in the diets of ruminants at the stated doses.

*Studies with cattle.* As noted previously, dietary Glu is extensively catabolized by the small intestine of ruminants. Thus, dietary supplementation with 0.2% MSG for 84 days did not have any adverse effects on calves [123]. Dairy cows could tolerate as much as 0.72% supplemental Glu in the diet for at least 21 days (the longest period of the study) [157], and 80 g Glu (as MSG) per day per cow (127 mg/kg BW/day) for 28 days [161]. Furthermore, Brake et al. [76] reported that the duodenal infusion of Glu (0.5 g/kg BW/day) for 6 days did not have any adverse effect on growing steers. The appropriate doses of dietary Glu that are indicated previously are safe in both young and adult cattle. Further systematic studies are required to determine safe Glu dose ranges for ruminants.

*Studies with sheep and goats.* Lambs receiving dietary supplementation with 0.5% MSG for 135 days exhibited normal rates of feed intake and growth [124]. Young rams receiving the intramuscular administration of Glu (7 mg/kg BW) every 3 days for 28 days were healthy and produced viable sperms [126,165]. Adult sheep could tolerate as much as 4% MSG in the diet for 64 days [125]. Furthermore, female goats receiving the intravenous administration of Glu (7 mg/kg BW) twice weekly (Monday and Friday) between mid-June and late September or between early June and early November exhibited normal reproductive function and physiological variables in their plasma [151,166]. There is no concern over any risk of ruminal and whole-body Glu metabolism in sheep or goats at nutritionally relevant doses [21,125,126,127,131,166]. The composition of skeletal muscle, white adipose tissue, or milk is not adversely affected by the use of Glu to feed these herbivores [128,129,130].

### 3.2. Gln Nutrition in Ruminants

#### 3.2.1. Gln Nutrition in Healthy Calves

The optimal development of the neonatal gut requires adequate Gln [30]. Although bovine milk, like porcine milk, contains a relatively large amount of Gln, the additional provision of Gln via supplementation may aid in enhancing the intestinal growth and maturation of calves, as reported previously for piglets [168]. This idea is supported by the following lines of experimental evidence. First, when fed a corn grain-, soybean meal-, and wheat bran-based weaning diet (containing 21.25% CP on a DM basis), the daily intravenous administration of 16 g Gln to Holstein calves between 35 and 49 days of age increased the villus height and crypt depth of the duodenum, jejunum, and ileum, as compared with the control group without Gln infusion [144]. The supplementation of Gln to the calves also augmented the concentrations of Gln, urea, and glucose in plasma, without affecting feed intake or BW gains [144]. An increase in Gln dose to 32 g/day did not result in additional benefits in calves [144]. Second, when fed liquid milk at 28 days of age (1 week before weaning) for 7 days and a pelleted starter ration at 35 days of age (the day of weaning) for 7 days, dietary supplementation with 2% Gln (on a DM basis) did not affect feed intake but increased daily gain, hip width, and body length in Holstein calves, compared with early-weaned calves not receiving Gln supplementation [145]. Interestingly, Gln supplementation shortened the time for calves to achieve a target starter intake of 1.0 kg/day (15 vs. 17 days), in comparison with the unsupplemented group [145]. Third, van Keulen et al. [143] conducted a study to define the effect of Gln in modulating the intestinal development of calves fed low versus high milk allowance, in which reconstituted whole milk (containing 26% fat and 26% protein on a DM basis) was fed at the rate of either 10% or 20% of arrival BW. Dietary supplementation with 1% Gln (on a DM basis) for 35 days between 4 and 39 days of age increased the villus height, width, and surface area of the duodenum, jejunum, and ileum as compared with the unsupplemented group in calves with high-level feed intake [143]. In contrast, Gln supplementation had no effect on calves with low-level feed intake [143]. These results indicate that the beneficial effects of dietary Gln on the small intestine depend on the availability of other nutrients. Thus, Gln is potentially an effective nutrient for the optimal growth and health of early-weaned calves through improving intestinal development, as previously reported for early-weaned piglets [168].

#### 3.2.2. Gln Nutrition in Calves with Diarrhea

Diarrhea is a major problem in calves reared under production conditions [120]. Calves with diarrhea exhibit poor intestinal absorption of nutrients, intestinal mucosal damage, growth suppression, and high rates of mortality [134]. Naylor et al. [132] conducted a clinical trial involving 21 diarrheic calves with rotavirus and coronavirus infections, which then received twice daily treatments of 2 L (i.e., 4 L/day) of an oral electrolyte solution containing 40 mM glycine (a positive standard treatment), 40 mM Gln in replacement of 40 mM glycine, or 400 mM Gln in replacement of 40 mM glycine, for 5 days. There were seven calves in each treatment group. The authors found that the addition of 40 and 400 mM Gln to an oral electrolyte solution without glycine may be beneficial for improving the intestinal health of diarrheic calves, based on the intestinal morphology and hydration status, as well as fecal water content and shape. The success rates of treatment were 7/7, 7/7, and 5/7, respectively, in the glycine, 40 mM Gln, and 400 mM Gln groups. Of note, calves in the 400 mM Gln group were depressed, likely due to elevated production and circulating levels of ammonia. These results indicate that although Gln may not be a better alternative to glycine in an oral electrolyte solution, the possibility remains that optimal doses of Gln and glycine may have a synergistic effect on the intestinal function of calves and should be evaluated in future studies.

In a subsequent study, Brooks et al. [134] evaluated the effects of high-glucose and Gln-supplemented oral solution on treating diarrheic calves. Calves were experimentally infected with enterotoxigenic *E. coli* and then received: no treatment (group C, *n* = 10), no treatment with intestinal samples being obtained immediately after infection for clinical assessment (group D, *n* = 10), treatment with a World Health Organization-type oral rehydration solution containing < 2% glucose (group W, *n* = 9), treatment with a World Health Organization-type oral rehydration solution containing high glucose (group N, *n* = 9), or treatment with a World Health Organization-type oral rehydration solution which contained high-glucose and glutamine (group G, *n* = 9). Results showed that the villus length and surface area of the small intestine were reduced to the greatest extent in calves receiving a World Health Organization-type oral rehydration solution containing < 2% glucose (group W), compared with those receiving high-glucose (group N) and high-glucose plus Gln (group G). Mean villus length (as a percentage of the control value) was 72.4% for group W, 85.8% for group N, and 85.8% for group G. Notably, diarrhea increased crypt depth throughout the intestine, and group G (calves given the Gln-supplemented solution) was the only group that exhibited a lower mitotic activity (the number of mitoses per crypt; an indicator of recovery from diarrhea) in the small intestine than that in group D. Collectively, these results indicate that the inclusion of Gln in a high-glucose oral rehydration solution can improve clinical recovery from diarrhea in calves.

In a similar experiment, Brooks et al. [133] reported that adding Gln to a high-glucose rehydration solution could result in the following benefits: (1) enhance plasma volume within 48 h after *E. coli*-induced diarrhea and sustain the improvement throughout the treatment period, and (2) correct the packed cell volume in the blood (the percentage of red blood cells in the blood) within 48 h after *E. coli*-induced diarrhea and sustain the benefit throughout the treatment period. Importantly, during the experimental period, calves in the two Gln-free rehydration solutions significantly lost 2.3–2.6 kg in BW, but calves treated with the Gln-supplemented solution did not lose weight.

Another study determined the effects of adding Gln to a standard oral electrolyte solution on ameliorating diarrhea in calves experimentally induced by enterotoxigenic *Escherichia coli* (0101:K99) infection [135]. There were four groups of calves (*n* = 6/group). Group 1 received a conventional oral electrolyte solution, group 2 received a high-glucose, Gln-free oral electrolyte solution, group 3 received a calcium- and magnesium-supplemented oral electrolyte solution without Gln, and group 4 received a calcium- and magnesium-supplemented oral electrolyte solution with Gln. The authors reported that the calcium- and magnesium-supplemented oral electrolyte solution with Gln resulted in improvement in more clinical parameters (skin tenting, mucous membrane color, mucous membrane moistness, warmth of extremities, and fecal consistency) than an oral electrolyte solution without Gln (i.e., groups 1, 2 and 3). Although only a small number of calves was used for this experiment, the clinical findings showed promising effects of Gln in treating *E. coli*-induced diarrhea in calves. Based on results from the published studies, Gln has been recommended for treating diarrhea in preweaning calves [130], as reported for weanling piglets [85].

#### 3.2.3. Gln Nutrition in Beef Cattle

Because of a short supply of pasture forages, especially high-quality ones, feedlot-finishing cattle are often fed high-grain diets to meet the energy demand for optimal growth performance. However, a significant problem in these animals is the occurrence of ruminal acidosis, particularly subacute ruminal acidosis. This is because (1) a large amount of bacterial endotoxin [e.g., lipopolysaccharide (LPS)] is released from the rumen [169]; and (2) the digestive tract (including the rumen and intestines) of cattle with grain-induced metabolic acidosis is compromised, which allows the translocation of LPS into the blood [170]. Thus, the efficient removal of free LPS from the circulation is essential to the health of feedlot cattle. In this regard, it is noteworthy that dietary supplementation with 1% Gln to grain-fed growing steers for 25 days increased the concentrations of LPS-binding protein in plasma by 200%, compared with the control group [136]. The underlying mechanisms are unknown, but the Gln-derived Glu may stimulate the synthesis of LPS-binding protein by enterocytes and the liver.

#### 3.2.4. Gln Nutrition in Dairy Cows

Based on the metabolic roles of Gln, Meijer et al. [119] proposed that Gln is a potentially limiting AA for milk production in dairy cows. However, several studies showed that the intra-abomasal infusions of 100 to 300 g Gln/day did not affect milk production by lactating cows or the nutrient composition of cow’s milk [70,97,98]. Similarly, dietary supplementation with rumen-protected Gln, which provided 100 g Gln [65] or 160 and 320 g Gln [137] to dairy cows did not affect milk yield. Likewise, the intra-abomasal infusions of 300 g Gln/day to lactating cows only slightly increased milk yield by 3% without affecting the nutrient composition of cow’s milk [87]. These studies should not be interpreted to indicate that Gln is sufficient for maximal milk production in cows fed a conventional diet containing ≤ 18% CP. It is possible that (1) the intra-abomasal infusions of 100–160 g unprotected Gln are ineffective in increasing the concentrations of Gln in plasma; (2) the intra-abomasal infusions of a larger amount of unprotected Gln (e.g., 200–300 g) reduces the intestinal absorption of some neutral AAs (e.g., glycine, tryptophan, threonine, phenylalanine, and tyrosine), compromising the protein nutritional status of cows and limiting milk protein synthesis by mammary epithelial cells; and/or (3) a co-deficiency of another AA in cows limits their lactation response to Gln alone. A major co-deficient AA in lactating cows is likely arginine, as reported for lactating sows [171]. Under favorable conditions that provide optimal ratios and amounts of all AAs (particularly arginine, lysine, and methionine), Gln may promote milk production by lactating cows.

Several studies have also shown that Gln has anti-inflammatory and antioxidative effects in lactating cows. For example, as reported for finishing steers [136], the intravenous infusions of 106 and 212 g Gln/day increased the concentrations of LPS-binding protein in the plasma of lactating cows [70]. Cows infused with 106 g Gln/day had greater concentrations of the serum amyloid A (an acute phase protein) in plasma on days 14 (+108%) and 21 (+106%) postpartum, compared with controls. Cows infused with 212 g Gln/day had greater concentrations of serum amyloid A on days 7 (+53%), 14 (+135%), and 21 (+235%) postpartum, compared with controls. These results indicate that Gln can regulate the production of acute phase mediators in dairy cows after parturition. Likewise, dietary supplementation with rumen-protected Gln (160 and 320 g/day) beneficially modulated immune responses and antioxidative defenses in lactating cows, particularly under heat stress conditions [67,137]. Similar results have been reported by other investigators [65]. This is consistent with the role of Gln in increasing the expression of heat-shock proteins and immunity in animals. Because of severe reductions in intramuscular concentrations of Gln in lactating cows [86], nutritional strategies are needed to ameliorate this metabolic disorder and improve milk production, particularly under stressful conditions.

Interestingly, dietary supplementation with rumen-protected Gln between 0 and 21 days post-partum has been reported to enhance lactation performance in dairy cows [139]. Specifically, compared with the control group (no Gln supplementation), adding 350 g Gln (in the rumen-protected form) daily per cow to an ~20 kg total mixed ration (containing 16.3% CP) for 21 days had the benefits of increasing (a) DM intake and milk yield by 17% and 12%, respectively, without affecting the concentrations of proteins or lipids in milk, and (b) the concentrations of total proteins, albumin, and glucose in plasma by 44%, 12%, and 24%, respectively, without affecting the concentrations of urea in plasma. In addition, compared with the control group, dietary supplementation with 350 g Gln/day to lactating cows decreased the concentrations of non-esterified fatty acids and β-hydroxybutyrate (possible indicators of a reduction in body fat mobilization) in plasma by 47% and 39%, respectively, and somatic cell counts in milk by 63%. Similar results were obtained for the dose of 250 g Gln/cow/day. In contrast, adding a lower daily dose of Gln (150 g/day) per cow for 21 days did not affect DM intake or milk yield, while increasing the concentrations of total proteins and glucose in plasma by 19% and 13%, respectively, and decreasing the concentrations of non-esterified fatty acids and β-hydroxybutyrate in plasma by 33% and 30%, respectively, and somatic cell counts in milk by 67%. Collectively, these results provide the proof-of-concept that increasing Gln availability in the abomasum and small intestine can improve milk production and mammary gland health in lactating cows.

#### 3.2.5. Gln Nutrition and Alleviation of Infections by Internal Parasites in Sheep

Studies with sheep have shown that Gln has a protective effect against the hepatic oxidation of AAs, particularly methionine, cysteine, and lysine [172], thereby increasing the availability of AAs for use by both the liver and extrahepatic tissues. In this regard, it is noteworthy that infection with internal parasites in ruminants (particularly grazing sheep and cattle) is a significant health problem, affecting both intestinal and hepatic metabolism (e.g., citrulline and albumin production, respectively). Because Gln and cysteine play important roles in immune responses and anti-inflammatory reactions, Hoskin et al. [69] determined the effects of intra-abomasal supplementation of 5 g Gln plus 1 g cysteine on the recovery of sheep from a 12-week subclinical *Trichostronglylus colubriformis* trickle infection. Infected sheep exhibited (1) increases in the total number of leukocytes and eosinophils in blood, nitrogen excreted in feces and urine, and the concentrations of total protein in plasma; and (2) reductions in BW gain and the concentrations of albumin in plasma without changes in feed intake. Supplementation with Gln plus cysteine beneficially increased nitrogen retention and decreased the number of circulating eosinophils. Thus, Gln can contribute to recovery and alleviating growth restriction in sheep infected with internal parasites; it is also an attractive potential therapeutic because there is widespread resistance to anthelmintics and because it is a non-toxic, environmentally safe alternative.

#### 3.2.6. Gln Nutrition and Alleviation of an Acid-Base Imbalance in Gestating Sheep

Using the ovine fetal alcohol spectrum disorders model, researchers studied the role of Gln in alleviating an acid/base imbalance in the mother and fetus [72,138,167]. In one study [167], pregnant sheep were assigned randomly to one of four groups: saline control, alcohol (1.75–2.5 g/kg BW), Gln (100 mg/kg BW), or alcohol + Gln. A chronic weekend binge drinking paradigm between gestational days (GD) 99 and 115 was utilized. Fetuses were surgically instrumented on GD 117 and studied on GD 120. Binge alcohol exposure caused maternal acidemia, hypercapnea, and hypoxemia, whereas fetuses were acidemic and hypercapnic. Alcohol exposure increased fetal arterial pressure and fetal brain blood flow, while reducing maternal uterine artery blood flow by 40%. Maternal Gln supplementation attenuated alcohol-induced maternal hypercapnia and fetal acidemia, while normalizing fetal brain blood flow. Furthermore, the administration of Gln to ewes, concurrent with alcohol administration, improved the profile of most AAs (including citrulline and arginine) in maternal and fetal plasma [138]. In another study [72], pregnant sheep were assigned randomly to four groups, saline control, alcohol (1.75–2.5 g/kg), Gln (100 mg/kg, three times daily), or alcohol + Gln. A weekend binge drinking model was followed where treatment was done 3 days per week in succession from GD 109–132 (normal term ~147), the equivalent of the third trimester in humans. Maternal alcohol exposure reduced fetal BW, height, length, thoracic girth, and brain weight, as well as the bioavailability of AAs in fetal plasma and placental fluids. Maternal Gln administration successfully mitigated alcohol-induced fetal growth restriction and improved the bioavailability of Gln and related AAs (e.g., glycine, arginine, and asparagine) in the fetal compartment [72]. Collectively, these findings show that Gln supplementation enhances AA availability in the fetus and prevents alcohol-induced fetal growth restriction.

#### 3.2.7. Gln Supplementation to Improve Ruminal Function in Lambs

Based on the finding that dietary supplementation with Gln improved gastrointestinal integrity and function in young pigs [85], studies were conducted with lambs to determine the role of Gln in their ruminal functions. Specifically, Wu et al. [141] reported that supplementing 0.5% or 1% Gln to a concentrate diet for 60 days enhanced the expression of claudin-1 and interleukin-10 (an anti-inflammatory cytokine) in ruminal epithelial cells by about 105% and 50%, respectively, in healthy 3-month-old male Hu lambs with an initial mean BW of 26.75 kg, compared with the control group. Furthermore, supplementation with 0.5% or 1% Gln increased pH, the ratio of acetate/propionate, and lipase activity in the ruminal fluid by about 4%, 25%, and 30% respectively, as well as the concentrations of interleukin-10 in serum by about 40% [141]. Collectively, Gln may play an important role in the development of ruminal fermentative activity, as well as the integrity, antioxidative capacity, and anti-inflammatory function of ruminal epithelial cells.

#### 3.2.8. Gln Supplementation to Alleviate Heat Stress in Goats and Lambs

Goats, like other ruminants, frequently experience heat stress during their life cycle in many parts of the world. These animals are mostly reared under an extensive management system with little or no shelter throughout the year. This problem can be exacerbated by high humidity, which can further reduce the growth and production performance of goats. At the molecular level, heat stress promotes the excessive generation of reactive oxygen species in animals (including ruminants) [173], which overwhelms the antioxidant defense capacity of the whole body, leading to oxidative stress. In a recent study, Ocheja et al. [68] determined the effect of the oral administration of Gln (0.2 g/kg BW in 10 mL water, once daily for 21 days) on Red Sokoto goats raised in the Northern Guinea Savannah zone of Nigeria that experienced intense solar radiant energy and high relative humidity for an extended period of time. The authors found that, compared with the control group receiving 10 mL water, Gln supplementation reduced rectal temperature, erythrocyte osmotic fragility, and malondialdehyde concentrations in serum, while increasing the activities of antioxidant enzymes (including superoxide dismutase, glutathione peroxidase, and catalase) in serum during weeks 1, 2, and 3. Similar results were recently reported for dietary supplementation with rumen-protected Gln (0.2 g/kg BW/day) to fattening lambs with heat stress for 45 days when fed total mixed rations containing 13.4% or 14.5% CP [140,142]. The underlying mechanisms are largely unknown but may include the effects of Gln-derived glutamate in the small intestine and stimulating the expression of antioxidative genes. Thus, Gln can mitigate heat stress-induced oxidative stress in goats and lambs during a hot-dry season.

#### 3.2.9. Gln Nutrition in Female Goats

Little is known about Gln nutrition in female goats. However, 2 mM Gln (six times its plasma concentration) is required for the optimal growth and development of caprine mammary epithelial cells and tissues [174], indicating that the normal concentrations of Gln in the plasma of goats (approximately 0.35 mM) are suboptimal for the maximal lactational performance of goats. Dietary supplementation with Gln, which is extensively converted into Glu in the rumen [14,19,20], may improve the reproductive performance of female goats as noted previously for the effect of Glu. However, direct evidence for the effect of Gln supplementation is lacking.

#### 3.2.10. Gln Nutrition in Rams

Gln is necessary for the nutrition and physiology of rams. However, little is known about the effects of Gln supplementation on their growth or fertility. Results of recent studies indicated that the proliferation, maturation, and function (e.g., fertilizing eggs) of ram spermatogonia depend on the presence of 20 mM Gln in the extracellular medium [113], indicating that the normal concentrations of Gln in the plasma of rams are inadequate for the maximal functions of sperm. Interestingly, there is evidence that Gln supplementation to semen extenders decreases lipid peroxidation, maintains the functional membrane and acrosomal integrity of ram sperm, and increases sperm viability and motility [113], indicating a role for Gln in ram fertility. Similar findings have been reported by Bucak et al. [112]. Dietary supplementation with Gln may improve reproductive performance in rams as was noted previously for the effect of Glu. However, direct evidence for the effect of Gln supplementation is lacking.

#### 3.2.11. Safety of Gln Supplementation in Ruminants

Factors that affect the safety of Glu supplementation in ruminants as noted previously also apply to Gln supplementation. As there is for an excessive amount of any AA in animals [1], there are reports of negative effects of Gln supplementation on the production performance of cattle if an incorrect dose is used [132]. This is likely related to elevated concentrations of both Gln and its metabolite (ammonia) in the blood that are toxic to the central nervous system [30].

*Studies with cattle.* As noted previously, dietary Gln is catabolized extensively by the bovine small intestine [78,87,175,176,177,178]. Dietary supplementation with 0.5 g Gln/kg BW/day to calves for 5 days did not result in any adverse effects, but a supplemental dose of 5 g Gln/kg BW/day is toxic [132]. Calves easily tolerated dietary supplementation with 1% Gln [178] or 1% AminoGut on a DM basis [179]. However, the oral administration of a 400 mM Gln solution to calves may result in brain abnormality [132]. Beef cattle fed a grain-based diet supplemented with 1% Gln for 25 days did not exhibit any adverse response [136]. Dairy cows tolerated intra-abomasal infusions of 300 to 320 g Gln/day, equivalent to approximately 1% of DM intake [70,88,97,98,137]. This safe level of Gln supplementation in cattle is similar to that for pigs [85] and poultry [180]. Appropriate doses of dietary Glu are safe in both young and adult cattle.

*Studies with sheep and goats.* Pregnant sheep receiving the intravenous administration of 300 mg Gln/kg BW/day for 23 days exhibited normal rates of feed intake and fetal growth [167]. Postweaning lambs receiving dietary supplementation with 1% Gln (equivalent to approximately 0.5 g Gln/kg BW/day) for 60 days did not show any adverse responses [141]. Ocheja et al. [68] reported that goats can tolerate the oral administration of 0.2 g Gln/kg BW/day for 21 days. Thus, appropriate doses of Gln are safe for sheep and goats.

## 4. Conclusions

Both Glu and Gln are abundant in feedstuff and animal proteins and in the free pool of AAs in tissues (e.g., skeletal muscle, liver, and brain) of animals, including ruminants. Results of recent studies have shown that although extracellular Gln is extensively utilized (primarily via degradation) by the ruminal microbes of both cattle and sheep, there is little catabolism of extracellular Glu by these cells due to negligible uptake by ruminal microbes. Thus, ruminal bacteria convert Gln into Glu plus ammonia and, intracellularly, use both AAs for protein synthesis. Microbial proteins and dietary Glu exit the rumen into the abomasum (where dietary proteins undergo limited hydrolysis) and then into the small intestine (where proteins undergo extensive extracellular degradation to release free AAs (including Glu and Gln) and small peptides for transport into enterocytes. Most dietary Gln escapes the underdeveloped rumen of preruminants (e.g., preweaning calves) into the small intestine. Within the enterocytes, Glu and Gln are extensively oxidized to provide ATP and are actively used to synthesize glutathione (a major antioxidant molecule) and AAs (alanine, ornithine, citrulline, arginine, proline, and aspartate), whereas Gln and aspartate are essential for purine and pyrimidine syntheses. Both Glu and Gln also beneficially regulate gut integrity and intestinal function. Although the small intestine takes up Gln from both its lumen and the systemic blood across the apical and basolateral membranes, respectively, of enterocytes, only luminal Glu enters these cells due to the lack of transporters in the basolateral membrane. Under normal feeding conditions, all of the diet- and rumen (microbial protein)-derived Glu and Gln are extracted by the small intestine in first-pass metabolism and, therefore, do not enter the portal circulation. Thus, de novo synthesis (e.g., from branched-chain AAs and α-ketoglutarate) plays a crucial role in the homeostasis of Glu and Gln in the whole body but may be insufficient for maximal growth performance and optimal health (particularly intestinal health). In support of this view, dietary supplementation with appropriate doses of Glu or Gln may improve the digestive, endocrine, and reproductive functions of ruminants to enhance their productivity. The responses of animals to supplemental doses are influenced by a plethora of factors, including feed composition, feeding frequencies, and management methods; environmental factors (e.g., low or elevated ambient temperatures, noise levels, and air pollution); the stage of the lifecycle (suckling, postweaning-finishing, lactating, pregnant, or adult); and pathological conditions (e.g., infection, injury, or inflammation). The oral (at least 0.5 g Glu/kg BW/day), intramuscular (7 mg Glu/kg BW/day), or intravenous (7 mg Glu/kg BW/day) administration of Glu is safe in young and adult ruminants. These animals can also safely tolerate either oral (e.g., 1 g/kg BW/day) or intravenous (e.g., 0.3 g/kg BW/day) administration of Gln. Further systematic studies are required to determine safe Glu and Gln dose ranges for ruminants. Both Glu and Gln are truly functional AAs in their nutrition. Supplementation with these two AAs to the diet provides a great potential for improving the health and production performance of ruminant species.

## Figures and Tables

**Table 2:** Estimated flow of L-glutamate and L-glutamine along the forestomach and the small intestine in adult, non-pregnant, and non-lactating sheep.

Variable	Adult Sheep (60 kg BW)	
	Glutamate	Glutamine
Feed intake (on an as-fed basis), ^a^ kg/day	1.20	1.20
Crude protein intake, g/day	140	140
Microbial and feed proteins entering the duodenum, ^b^ g/day	95.2	95.2
Nitrogen leaving the rumen as ammonia and reentering the rumen via N recycling for microbial protein synthesis, ^c^ g/day	18.2	18.2
Total microbial and feed proteins in the small intestine, g/day	113.4	113.4
Digestible microbial and feed proteins in the small intestine, ^d^ g/day	96.4	96.4
Glu or Gln released from digestible microbial and feed protein, ^e^ g/day	7.71	5.30
Glu or Gln entering the portal vein (100% extraction by the small intestine), g/day	0	0
Glu or Gln catabolism in the whole body, g/day	51.5 ^f^	46.7 ^g^
Glu or Gln required for protein deposition (maintenance), g/day	0	0
Glu or Gln oxidation in the portal-drained viscera, g/day	7.71	5.30
Endogenous synthesis of Glu and Gln, ^h^ g/day	43.8	41.4
% of dietary crude protein used for Glu and Gln synthesis ^i^	31.3	29.6

^a^ the dietary content of crude protein, glutamate, and glutamine (on an as-fed basis) is 11.7%, 0.94%, and 1.10%, respectively [53]. ^b^ 68% of the dietary crude protein intake [30]. ^c^ 13% of the dietary nitrogen intake [30]. ^d^ the true digestibility of proteins in the small intestine is 85% [30]. ^e^ it is assumed that microbial protein and feed protein account for 90% and 10% of the total proteins, respectively. The content of glutamate and glutamine in sheep ruminal microbes (g/100 g protein) is 8.02% and 5.11%, respectively, whereas the content of glutamate and glutamine in the feed protein for sheep (g/100 g protein) is 7.85% and 9.02%, respectively. ^f^ based on the rate of whole-body glutamate catabolism in adult sheep (0.596 mg/min/kg body weight) [64]. ^g^ Based on the rate of whole-body glutamine catabolism in adult sheep (0.222 mmol/h/kg body weight) [58]. ^h^ calculated as Glu or Gln catabolism in the whole body—Glu or Gln oxidation in the portal-drained viscera. ^i^ calculated as (the endogenous synthesis of Glu or Gln)/Crude protein intake × 100%.

**Table 3:** Net uptake or net release of amino acids by the liver of lactating cows ^a^.

Amino Acid	Net uptake (+) or Net Release (–) by the Liver	Amino Acid	Net Uptake (+) by the Liver
Ala	+11	Leu	+2.1
Arg	+4.9	Lys	+2.2
Asn	+17	Met	+9.9
Asp	+2.4	Phe	+12
Citrulline	+0.1	Pro	+6.5
Gln	+5.7	Ser	+19
Glu	–24	Thr	+5.3
Gly	+15	Trp	+4.2
His	+4.8	Tyr	+11
Ile	+1.9	Val	+1.3

Adapted from Doepel et al. [87]. Values are % of the portal vein flux, unless specified otherwise. ^a^ Cows consumed 19.4 kg of dry matter per day and produced 16.5 kg of milk per day.

**Table 4:** Production of CO_2_, ornithine, citrulline, and arginine from glutamine by enterocytes of postnatal cattle and sheep.

Animals	Production of Metabolites from Glutamine (nmol/mg DNA/30 min)			
	CO_2_	Ornithine	Citrulline	Arginine
Brahman cattle (*n* = 5 per age group)				
2-day-old calves	3903 ± 228 ^a^	15.8 ± 1.4 ^a^	149 ± 13 ^a^	185 ± 20 ^a^
7-day-old calves	3725 ± 301 ^a^	15.0 ± 1.2 ^a^	132 ± 16 ^a^	169 ± 22 ^a^
6-month-old calves	906 ± 54 ^b^	5.13 ± 0.09 ^b^	44.7 ± 2.5 ^b^	9.22 ± 0.61 ^b^
24-month-old beef cattle	410 ± 291 ^c^	3.76 ± 0.07 ^c^	30.2 ± 1.1 ^c^	2.30 ± 0.14 ^c^
Suffolk sheep (*n* = 6 per age group)				
0-day-old lambs	4582 ± 170 ^a^	21.4 ± 0.79 ^a^	205 ± 9.0 ^a^	390 ± 13 ^a^
3-month-old lambs	1359 ± 51 ^b^	10.6 ± 0.35 ^b^	86.8 ± 3.8 ^b^	40.2 ± 1.2 ^b^
24-month-old (non-pregnant and non-lactating) ewes	406 ± 16 ^d^	6.12 ± 0.19 ^d^	39.0 ± 1.3 ^d^	2.84 ± 0.15 ^d^
24-month-old pregnant ewes	493 ± 19 ^c^	7.56 ± 0.22 ^c^	48.7 ± 1.6 ^c^	3.72 ± 0.20 ^c^

Adapted from Wu et al. [74]. The Krebs bicarbonate buffer (pH 7.4) for the incubation of enterocytes contained 5 mM D-glucose and 2 mM L-glutamine. ^a–d^: Within a column for each animal species (e.g., cattle or sheep), means not sharing the same superscript letters differ (*p* < 0.05).

**Table 5:** Effects of administration of L-glutamate on metabolism and production performance in preruminants and ruminants ^a^.

Animal	Basal Diet	Administration of L-Glutamate	Responses to L-Glutamate	Reference
Steer calves	A semi-purified diet with 97% of nitrogen from urea	Dietary supplementation with 0 or 3% Glu for 21 days	↑ ruminal production of fatty acids with ≥4 carbons	Oltjen et al. [122]
Dairy valves	Milk replacer with 24.8% CP and 19.1% fat	Dietary supplementation with 0 or 0.3% Glu (on a DM basis) for 8 weeks between 3 and 59 days of age	No changes in feed intake, weight gain, or plasma concentrations of urea and proteinogenic AAs	Ahangarani et al. [148]
Dairy calves	Fresh whole milk (2.3 kg twice daily) plus a starter diet (0 to 21 days of age); alfalfa-based diet after weaning (21 days of age); 18% CP in solid diets	Either 0 or 0.2% MSG between 0 and 21 days of age; and between day 21 (weaning) and day 84 of age	↑ 321% for solid feed intake in week 3 of age; ↑ 248%, 107%, and 40% feed intake in weeks 1, 2, and 3 postweaning, respectively	Waldern and van Dyk [123]
Dairy calves	Same as above, except that the weaning age was 28 days, but not 21 days	Either 0 or 0.2% MSG between 0 and 28 days of age; and between day 28 (weaning) and day 42 of age	↓ weight loss (91 vs. 45 g per day in the first week after weaning	Waldern and van Dyk [123]
Growing steers	Soybean hull-based diets; BW of cattle = 259 kg	Duodenal infusions of either 0 or 133 g Glu and 1391 g raw cornstarch per day for 6 days	↑ starch digestion in the small intestine	Brake et al. [76]
Growing steers	Soybean hull-based diets; BW of cattle = 179 kg	Duodenal infusions of either 0 or 121 g Glu and 1460 g raw cornstarch per day for 42 days	No effect on starch digestion in the small intestine	Acharya et al. [156]
Neonatal lambs	A diet consisting of lucerne and concentrates	Either 0 or 0.5% MSG beginning at 18 days of age	↑ feed intake and weight gain between 90 and 135 days of age	Galgan and Russell [124]
Adult sheep	Ammoniated barley straw; or pelleted lucerne	Either 0 or 1% MSG for 64 days; or 0.5% and 4% MSG for 64 days	↑ feed intake by 10%, 16%, or 40%, respectively	Colucci and Grovum [125]
Rams	Alfalfa hay and corn-based diet	im administration of Glu (0 or 7 mg/kg BW every 3 days) for 28 days	↑ sperm production by 51%; ↑ GnRH secretion	Meza-Herrera [126]
Female goats	A mixed diet ^b^	im or iv administration of Glu (0 or 7 mg/kg BW twice weekly) From mid-June to late October	↑ the onset of puberty without affecting BW	Torres-Moreno et al. [127]
Lactating cows	Soybean meal (25%)-based diet	Dietary supplementation with 0, 0.24, 0.48, or 0.72 % Glu as 5, 10, and 15% MSG by-product, respectively	↑ milk production by 15, 22, and 33%, respectively	Nakanishi et al. [128]
Sheep with heat stress	Total mixed ration with 14.7% CP	Dietary supplementation with 0 or 3 g Glu/head/day for 90 days	↑ rumen fermentation; ↑ antioxidative capacity; ↑ growth performance	Li et al. [129]
Lactating cows	Total mixed ration with 16.5% CP (prepartum) and 18.3% CP (postpartum)	Dietary supplementation with 0 or 1.54% Glu (prepartum) plus 1.14% Glu (Postpartum) as MSG	↑ feed intake; ↑ digestive capacity; ↓ losses of whole-body fat and protein after calving	Hisadomi et al. [130]
Female goats	Total mixed ration	Dietary supplementation with 0 or 1 g MSG/kg BW for 23 days	↑ feed intake; ↑ ruminal movement; ↑ rumination; ↑ ovarian follicle number; ↑ intraovarian blood flow	Soares et al. [131]

^a^ in all these studies, the administration of Glu or MSG had no adverse effect on the animals. ^b^ goats were fed twice daily, with alfalfa hay (14% CP) in the morning and corn silage (8.1% CP) plus corn grain (11.2% CP) in the afternoon. BW, body weight; CP, crude protein; Glu, L-glutamate; GnRH, gonadotropin hormone-releasing hormone; MSG, monosodium glutamate; im, intramuscular; iv, intravenous; SI, small intestine; ↑ increase or improve; ↓ decrease.

**Table 6:** Effects of administration of L-glutamine on metabolism and production performance in preruminants and ruminants ^a^.

Animal	Basal Diet	Administration of L-Glutamine	Responses to L-Glutamine	Reference
Healthy calves	Grain-based diet with 21.25% CP	iv infusion of Gln (0 or 16 g/day) for 14 days between 35 and 49 days of age	↑ villus height and crypt depth in the duodenum, Jejunum, and ileum; ↑ plasma glucose Conc.	Hu et al. [144]
Healthy calves	Liquid milk at 28–34 days of age; grain- based diet between day 35 (weaning) and day 42 of age	Dietary supplementation with 0 or 2% Gln (on a DM basis) for one week both before and after weaning	↑ daily weight gain; ↑ adaptation to weaning ration; ↑ hip width and body length	Wickramasinghe and Appuhamy [145]
Healthy calves	Reconstituted whole milk with 26% CP and 26% fat (on a DM basis); daily feed intake at 10 or 20% of initial BW	Dietary supplementation with 0 or 1% Gln (on a DM basis) for 35 days between 4 and 39 days of age	↑ villus height, width, and surface area in duodenum and jejunum of calves with high-level feed intake; no effect in calves with low-level feed intake	van Keulen et al. [143]
Calves with diarrhea	Cow’s milk	Oral electrolyte solution with 0 or 40 mM Gln	↑ morphology of the SI; ↑ hydration status	Naylor et al. [132]
Calves with diarrhea	Cow’s milk	Oral electrolyte solution with no or high Gln concentration	↑ hydration status; ↑ recovery from diarrhea; ↓ weight loss by 2.3–2.6 kg	Brooks et al. [133]
Calves with diarrhea	Cow’s milk	Oral electrolyte solution with no or high Gln concentration	↑ morphology of the SI; ↑ recovery from diarrhea	Brooks et al. [134]
Calves with diarrhea	Cow’s milk	Oral electrolyte solution with no or high Gln concentration	↑ hydration status; ↑ recovery from diarrhea	Pal and Pachauri [135]
Beef cattle (feed-lot)	Grain-based diet	Dietary supplementation with 0 or 1% Gln for 25 days	↑ clearance of endotoxin from the blood	Jin et al. [136]
Dairy cows	Concentrates-based diet with 18% CP	Intra-abomasal infusion of Gln (0 or 300 g/day) for 21 days	↑ milk yield by 3%	Doepel et al. [87]
Dairy cows	Concentrates-based diet with 18% CP	iv infusion of Gln (0, 106, or 212 g/8 h per day) for 7 days	↑ production of acute-phase protein by the liver	Jafari et al. [70]
Dairy cows	Concentrates-based diet with 18% CP	Rumen-protected Gln (0, 160, or 320 g/day) for 60 days	↑ immune response; ↑ anti-oxidative capacity	Caroprese et al. [137]
Sheep with trickle infection	Roughage-based diet	Daily intra-abomasal infusion of Gln (0 or 5 g) and cysteine (0 or 1 g) for 12 weeks	↑ N retention in the body; ↓ systemic infection	Hoskin et al. [69]
Gestating sheep	Roughage-based diet with 12% CP	iv infusion of Gln (0 or 100 mg/kg BW 3 times daily) for 23 days to ewes with alcohol-induced acidosis	Prevents alcohol-induced acidosis in fetus; prevents alcohol-induced increase in fetal brain blood flow	Washburn et al. [138]
Gestating sheep	Roughage-based diet with 12% CP	iv infusion of Gln (0 or 100 mg/kg BW 3 times daily) to ewes with alcohol-induced acidosis between 109 and 132 days of gestation	Prevent alcohol-induced acid-base imbalance and alcohol-induced IUGR	Sawant et al. [72,167]
Goats with heat stress	Corn offals and hays	Oral Gln (0 or 0.2 g/kg BW, once daily) for 21 days	↓ rectal temperature; ↓ erythrocyte fragility; ↓ oxidative stress	Ocheja et al. [68]
Lactating cows	Total mixed ration with 16.3% CP	Dietary supplementation with rumen-protected Gln (0, 250 or 350 g/cow/day) for 21 days	↑ DM intake; ↑ milk yield; ↑ plasma levels of glucose, and proteins; ↓ plasma BHB and NEFA; ↓ SCC in milk	Nemati et al. [139]
Lactating cows	Total mixed ration with 16.3% CP	Dietary supplementation with rumen-protected Gln (0 or 150 g/cow/day) for 21 days	NC for DM intake or milk yield; ↑ plasma levels of glucose and proteins; ↓ plasma BHB and NEFA; ↓ SCC in milk	Nemati et al. [139]
Lambs with heat stress	Total mixed rations with 13.4–14.5% CP	Dietary supplementation with rumen-protected Gln (0 or 0.2 g/kg BW per day) for 45 days	↑ antioxidative capacity; ↓ cortisol in serum; ↓ systemic NO production	Feyz et al. [140]
Lambs (healthy)	High-concentrate diet with 13.3% CP	Dietary supplementation with 0, 0.5 or 1% Gln for 60 days	↑ expression of claudin-1 and interleukin-10 in the ruminal epithelium; ↑ Ac/Prop ratio and lipase activity in ruminal fluid	Wu et al. [141]
Lambs with heat stress	Total mixed rations with 13.4–14.5% CP	Dietary supplementation with rumen-protected Gln (0 or 0.2 g/kg BW per day) for 45 days	↑ antioxidative capacity, as indicated by reduced	Mohamadzadeh et al. [142]

Ac/Pro, acetate/propionate; BHB, β-hydroxybutyrate; BW, body weight; Conc, concentration; CP, crude protein; DM, dry matter; Gln, L-glutamine; IUGR, intrauterine growth restriction; iv, intravenous; NC, no change; NEFA, non-esterified fatty acids; NO, nitric oxide; SCC, somatic cell counts; SI, small intestine; ↑ increase or improve; ↓ decrease.

## Data Availability

Not applicable.
